# Effect of Phyto-Assisted Synthesis of Magnesium Oxide Nanoparticles (MgO-NPs) on Bacteria and the Root-Knot Nematode

**DOI:** 10.1155/2022/3973841

**Published:** 2022-08-08

**Authors:** Azhar U. Khan, Masudulla Khan, Azmat Ali Khan, Aiman Parveen, Sajid Ansari, Mahboob Alam

**Affiliations:** ^1^School of Life and Basic Sciences, Department of Chemistry, SIILAS Jaipur National University, Jaipur 302017, Rajasthan, India; ^2^Botany Section, Women's College, Aligarh Muslim University, Aligarh-202002, Uttar Pradesh, India; ^3^Pharmaceutical Biotechnology Laboratory, Department of Pharmaceutical Chemistry, College of Pharmacy, King Saud University, Riyadh 11451, Saudi Arabia; ^4^Department of Botany Aligarh Muslim University, Aligarh 202002, Uttar Pradesh, India; ^5^Department of Safety Engineering, Dongguk University, 123 Dongdae-ro, Gyeongju-si 780714, Gyeongsangbuk-do, Republic of Korea

## Abstract

The root-knot nematode was examined using magnesium oxide nanoparticles (MgO-NPs) made from strawberries. The biologically synthesized MgO-NPs were characterized by UV, SEM, FTIR, EDS, TEM, and dynamic light scattering (DLS). Nanoparticles (NPs) were examined using scanning electron microscopy (SEM) and transmission electron microscopy (TEM) and shown to be spherical to hexagonal nanoparticles with an average size of 100 nm. MgO-NPs were tested on the root-knot nematode *M. incognita* (Meloidogynidae) and the plant pathogenic bacteria *Ralstonia solanacearum*. The synthesized MgO-NPs showed a signiﬁcant inhibition of *R. solanacearum* and the root-knot nematode. MgO-NPs cause mortality and inhibit egg hatching of second-stage juveniles (J2) of *M. incognita* under the in vitro assay. This study aims to examine the biological activity of biogenic MgO-NPs. The findings marked that MgO-NPs may be utilized to manage *R. solanacearum* and *M. incognita* and develop effective nematicides. In addition, the antioxidant capacity of MgO-NPs was determined by using 2, 2-diphenyl-1-picryl-hydrazyl-hydrate (DPPH).

## 1. Introduction

Commercial agriculture mainly relies heavily on chemical pesticides to protect crops against pathogens and pests. Different approaches are used to mitigate plant diseases. Nanotechnology is an emerging significant area in modern science [1]. Nano is a Greek word that corresponds to one-billionth; hence, nanotechnology functions with one-billionth of a meter-sized material. In recent years, nanotechnology has been extensively utilized in the production of antimicrobials against pathogenic bacteria that are damaging to humans, crops, and animals. The application of nanomaterials for the improvement of growth and production of crops and plant disease control is the global hot topic of research [2]. Since its significant recent development, researchers have been fascinated by the synthesis of metallic carbon-based and polymeric nanomaterials and their application for effective pathogenic plant disease management [3, 4]. Moreover, drug delivery and sustained release with increased bioavailability can be accomplished using nanoparticles (NPs) in a cost-effective strategy. As described in the literature [5, 6], a number of variables, such as airflow, breathing rate, lung volume, and particle size, affect the delivery of NPs to the lung as well as their distribution and deposition. Plant-based synthesis of nanoparticles is a simple, environmentally safe, economical, and safer approach for human use [7]. Plants or plant extracts are utilized as reducing and capping agents in the manufacture of nanomaterials [8, 9]. This is a more straightforward biological method with additional advantages [10]. The plant extract reduces the magnesium and acts as a stabilizing agent [11–13]. Magnesium (Mg) is responsible for regulating various biochemical and physiological processes in plants and is hence considered an essential element, and it is also a crucial part of plant defense mechanisms during abiotic stress [14]. Due to its mobility inside phloem, Mg ensures the transport of photosynthesis in phloem, enzyme activation during protein biosynthesis, and synthesis of chlorophyll in actively growing areas of plants [15]. Magnesium oxide nanoparticles (MgO-NPs) have the potential to inhibit bacterial growth [2, 16] including antioxidant, anticancer, and anti-inflammatory properties [17, 18]. MgO-NPs could penetrate the bacterial cell wall and could kill bacteria. The effect of MgO-NPs depends on particle size [2, 16, 19]. Sundrarajan et al. [20] observed that MgO-NPs had antibacterial activities against *S. aureus* and *E. coli* bacteria. At the present time, agriculture is facing challenges to achieve food demand. The worldwide population could likely reach 9.7 billion by 2050, so an annual increment of 2.4% is necessary to achieve the food demand. High loss in agriculture occurs due to diseases caused by pathogens and pests. *R. solanacearum* is a plant pathogenic, highly destructive Gram-negative bacterium that causes wilt disease to more than 100 crop plant species. It causes high crop yield losses worldwide [21]. Despite the fact that MgO nanoparticles kill germs and keep fungi at bay, there are not many studies on root-knot nematodes in the literature [22]. In modern pest management, pesticides such as insecticides, fungicides, and herbicides are frequently used. Although pesticides have many advantages, such as their ease of use, quick action, and dependability, they can also harm creatures that are not their intended targets, encourage the regrowth of insect populations, and develop pest resistance [23]. New concepts and agricultural products with significant promise for resolving the aforementioned problems have been developed as a result of nanotechnology. Nanoparticles having desirable characteristics, such as form, pore size, and surface properties, have been developed by material scientists. The potential for a new generation of insecticides and other activities for managing plant diseases will significantly expand as agricultural nanotechnology advances [24], leading to high crop output. *Phytoparasitic nematodes* (PNs) are soil-borne, obligate biotrophs and cause enormous yield losses to crops worldwide per year [21]. *Meloidogyne incognita* (Meloidogynidae) impacts the cultivation of the brinjal crop. *Meloidogyne* spp. is a sedentary endoparasite whose females grow inside the root of host plants. The second-stage juvenile (J2) is the infective stage of *Meloidogyne* spp., which punctures the host root plants and feeds on the root cells. They induce cell division and hypertrophy, and the formation of galls occurs in the roots [21]. This study was conducted to test the effects of biosynthesized MgO-NPs on root-knot nematode *R. solanacearum* as well as their antioxidant capacity.

## 2. Materials and Methods

### 2.1. Materials

Strawberry seeds were collected from fruit Mandi Sanganer, Jaipur, India. All chemical materials used were of analytical grade and purchased from Merck India. They were used without additional purification as received. All glassware was cleaned with acetone and then rinsed with double distilled water and dried before usage.

### 2.2. MgO-NP Synthesis

The strawberry powder was made from 1.6 gm of dried strawberry seeds. This fine powder of seeds was boiled in deionized water (100 ml) for 30 min and cooled at ambient temperature. The resulting solution was passed through the Whatman filter paper. The filtered extract was kept in a refrigerator for further thermosynthesis of the nanoparticles. Add dropwise 30 ml of an aqueous solution of magnesium nitrate (0.1 M) to 70 ml of strawberry extract in a 250 ml flask with magnetic stirring at 50–60°C. During the reaction, the color change is observed from transparent to white on vigorous stirring for 3 hrs. Finally, the NPs are collected and dried at 40°C in a China dish for 8 h before being calcined to produce biosynthesized MgO-NPs.

### 2.3. Characterization of MgO-NPs

Morphology, microstructure, and elemental composition of the magnesium oxide-NP sample were observed by using a scanning electron microscope (SEM : JEOL JSM 6510LV) provided with an energy dispersive X-ray analyzer (EDX). The structure and particle size of synthesized magnesium oxide-NPs were analyzed by transmission electron microscopy (TEM) (TEM : JEM-2100). Biosynthesized MgO-NPs were finally confirmed by spectral studies such as SAED. The crystalline structure of synthesized MgO-NPs was analyzed by using an X-ray diffractometer (XRD). FTIR : Nicolet iS10 (Fourier transform infrared; FTIR) spectroscopy with a wave number range of 350–4000 cm^−1^ was used to investigate the bond types in MgO-NPs. The size distribution of particles and the zeta potential (*ζ*) were examined using Malvern Instruments Zetasizer Nano ZS, which measured dynamic fluctuations in the light scattering intensity produced by the Brownian motion of the particles.

### 2.4. Antimicrobial Activity

The antibacterial activity of the biomanufactured magnesium oxide nanoparticles against *R. solanacearum* bacteria was evaluated using the disk diffusion method. The following gradients were used to make the nutrient agar medium: peptone (5.0 g), beef extract (3.0 g), and sodium chloride (5.0 g) in 1000 mL of distilled water. Agar (15.0 g) was added to the medium after the pH was adjusted to 7.0. The sterilization of the prepared medium took 20 min at 121°C in an autoclave. These sterilized nutrient agar media were poured onto Petri dishes. After solidification of the media, the bacterial culture was administered on the solid surface of the media and swabbed with a sterile cotton swab. The sterile paper discs (8 mm) were impregnated with sample solutions containing 10, 20, 30, 40, and 50 *μ*g/ml MgO-NPs. The impregnated discs were then set on inoculated agar and incubated at 37°C for 24 hrs. The zone of inhibition (ZOI) was calculated after incubation by subtracting the disk diameter from the total inhibition zone diameter and comparing it to the reference drug. Lower concentrations of MgO-NP in nutritional agar had less antibacterial action against *R. solanacearum* than higher concentrations. The sterilized water was added to bring the bacterial suspension to 10^5^ colony-forming units (cfu) per mL. In comparison to the control, minimum inhibitory concentration (MIC) is defined as the concentration at which no growth is seen. The noninhibitory concentration (NIC) is the lowest concentration at which normal observable growth can occur [4, 7].

### 2.5. Nematode Mortality Bioassay

**Figure 1:**
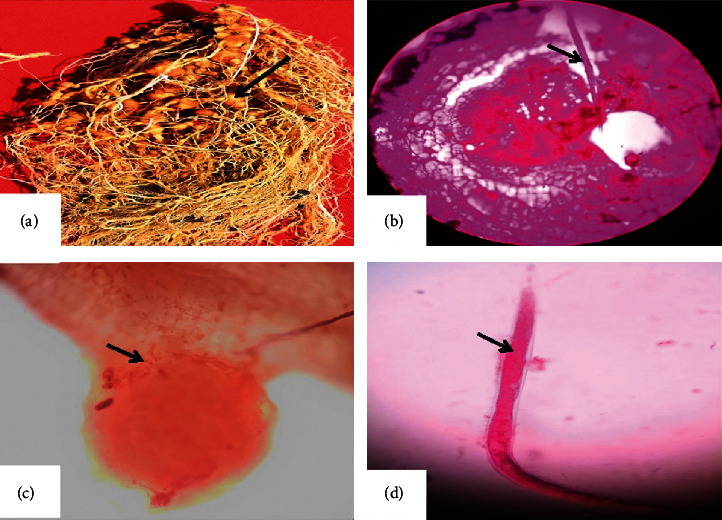
(a) Infected roots with *M. incognita*. (b) *M. incognita* in roots. (c) Egg mass of *M. incognita.* (d) Juvenile of *M. incognita.*

To detect the efficacy of MgO-NPs on the mortality of *M. incognita*, 20 ml suspension was prepared with 5, 50, and 100 *μ*g/ml of MgO-NPs and 15 ml of distilled water and placed in each Petri plate separately. 20 freshly hatched J2 were placed in each Petri plate. The plates containing freshly hatched J2 and MgO-NP solution were allowed to incubate at 25 ± 1°C, and the effect on mortality was observed after 24 and 48 h intervals. From infected roots, we collected the egg mass and J2 juvenile of *M. incognita* (Figure 1).

### 2.6. Egg Hatching Assay

50 *μ*g/ml and 100 *μ*g/ml suspensions (5 ml) were mixed in 15 ml of distilled water, and 20 ml was placed in each Petri plate to perform the hatching assay. In each Petri plate, 10 egg masses were inserted. As a control, the Petri dish with ten egg masses and 20 ml of double distilled water was employed. For 24 and 48 h, an influence on nematode hatching was detected. The number of hatched juveniles from eggs was counted by using the microscope [7].

### 2.7. Antioxidant Capacity Test with DPPH

DPPH was used to evaluate the antioxidant activity of MgO-NPs using a modified version of the method previously applied to evaluate the antioxidant properties of other nanoparticles [25]. In brief, a 50 *μ*L aliquot of different concentrations of MgO-NPs (75, 150, 300, and 500 *μ*g/mL) was added to 50 *μ*L mixture of 0.1 mM DPPH in methanol and incubated at room temperature for 10 minutes. Visual examination of the reaction between methanolic solutions of DPPH and MgO-NP reveals a color change from deep violet to colorless or pale yellow (in the presence of MgO-NP). Methanol and methanolic DPPH were used as negative and blank controls, while ascorbic acid was used as a reference compound to compare the antioxidant capacity of the nanoparticles. The absorbance was noted at 517 nm using a microplate reader. Different concentrations of MgO-NPs were evaluated against DPPH in triplicate. The radical scavenging activity or the other term inhibition percentage was calculated using the following equation:

(1) $$\begin{array}{c} \text{\% Inhibition}=\frac{A_{c}-A_{s}}{A_{c}}\times 100. \end{array}$$

Where *A*_c_ and *A*_s_ represent the absorbance of the control and the absorbance of the sample or standard sample, respectively.

### 2.8. Statistical Analysis

The data were analyzed with MS Excel and *R* software, and statistical significance was set as *p* < 0.05.

## 3. Results

### 3.1. UV-Vis Spectrophotometry

**Figure 2:**
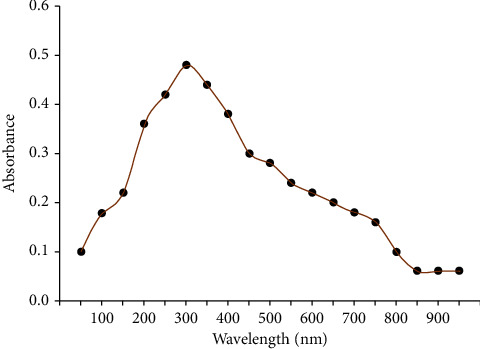
UV-Vis spectrum exhibiting maximum absorption at 290 nm because of presence of MgO-NPs.

The arrangement and size of metallic nanoparticles have a big impact on their characteristics. The MgO-NP solution UV-Vis absorption spectrum displays a significant absorption band at 290 nm (Figure 2). A wide absorption peak between 270 and 320 nm affirmed the nanorange dimensions of MgO particles. It corresponds to the MgO nanosphere dipole resonance [13]. The outcome of UV-Vis noticeably demonstrates that the reductive biomolecules in the strawberry extract were capable of bioreduction, resulting in the formation of MgO-NPs. Strawberry biomolecules' functional groups-C=O-, -C=C-, -C-O-C, and-C-O-have been proposed to perform as reducing agents in an environmentally friendly manner [26]. Excitation of the electron from oxygen 3-C corner atoms could be a cause of a wide absorption band [27].

### 3.2. Structural Characteristics

**Figure 3:**
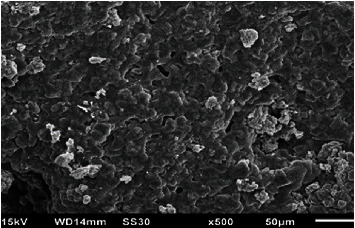
SEM micrograph of MgO-NPs.

**Figure 4:**
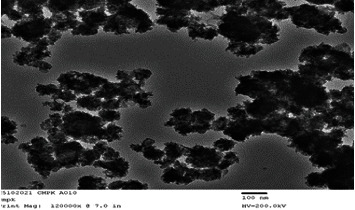
TEM micrograph of MgO-NPs.

**Figure 5:**
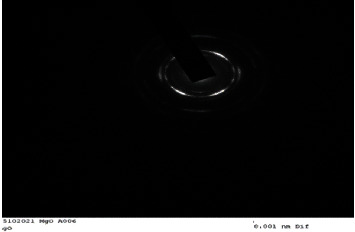
SAED pattern for the biogenic MgO-NPs.

The size and morphology of biosynthesized MgO-NPs were investigated using SEM and TEM imaging (Figures 3 and 4). The TEM micrograph recorded by the TEM grid (Figure 4) confirmed single-crystalline nature of NPs. The nanoscale size range of MgO-NPs was demonstrated by using TEM micrographs, with most of the NPs being spherical with an average diameter of 100 nm and a few particles having a significant scale range. The selected area electron diffraction (SAED) study affirmed the single-crystalline structure of synthesized MgO-NPs as shown in Figure 5. The particles are well distributed and have a large surface area-to-volume ratio.

### 3.3. Elemental Composition of MgO-NPs

**Figure 6:**
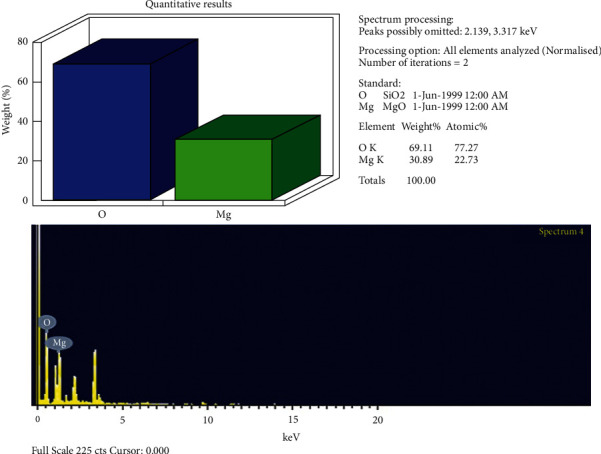
EDX graph of synthesized MgO-NPs.

The EDX analysis was used to verify the presence of magnesium in synthesized nanoparticles. The spectrum of EDX demonstrated elemental composition (Mg and O), with the weight and atomic percentage of each element in the sample (Figure 6) [28].

### 3.4. MgO-NPs' Diffraction Pattern

**Figure 7:**
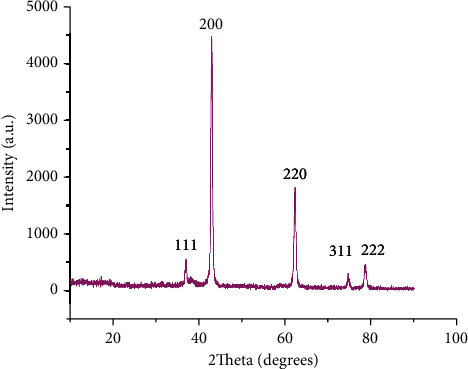
XRD spectrum of MgO-NPs.

XRD of biosynthesized MgO-NPs showed the crystalline structure and diffractogram as displayed in Figure 7, and the diffraction pattern exhibits peaks corresponding to the refraction planes (111), (200), (220), (311), and (222). The peaks in the XRD pattern match with those in the standard reference file (JCDPS file no ^#^ 39–7746 and 75–0447), indicating the formation of a hexagonal MgO phase [19, 29].

### 3.5. FTIR Spectrum

**Figure 8:**
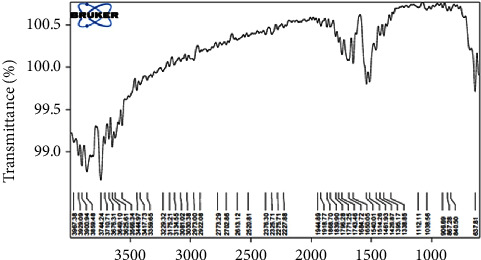
FTIR spectrum of MgO-NPs.

In the FTIR spectrum of MgO-NPs shown in Figure 8, the stretching vibration of the O–H group in alcohol is induced by the protective contact of the hydroxyl group of the phytochemicals in the extract with the MgO-NPs that show a wide absorption band centered at 3744 cm^−1^ (Figure 8). Strawberries have citric acid, ascorbic acid, and phenols [26, 30]. The modest double peaks at 2973 and 2922 cm^−1^ are thought to be C-H stretching vibrations in the CH2 group, which are found in phytochemicals. The presence of the N-H bond originating from the bending vibrational mode in aromatic amine is shown by the split sharp peak at 1540 cm^−1^. The aromatic amine may have been produced in the reaction of alkynes and alkanes with nitrate from the Mg(NO_3_)_2_.6H_2_O precursor in the strawberry extract. Changes in the mixture during the MgO-NPs formation stage, such as oxidation, reduction, or degradation, allow for this transformation. Mg-O vibration is ascribed to a peak at 1338 cm^−1^, whereas diagnostic bonds C-O-C and C-O are assigned to the modest peaks at 1038 and 906 cm^−1^. The formation of spherical MgO-NPs is shown by the peak of approximately 637 cm^−1^, following the results of SEM, TEM, and XRD. The literature and IR spectrum of the NPs discussed above reveals the nature of phytochemicals and their role in nanoparticle synthesis and their stability. According to investigation [31–33], strawberries and their seeds contain a variety of phenolic chemical components, with anthocyanidins, procyanidins, phenolic acids, and flavonoids being among the most important. The concentration and composition of these phytochemicals, on the other hand, vary by variety, growth place, and growing season. Despite the presence of many other phytocomponents in strawberries and their seeds, quercetin-3-glucuronide, belonging to the flavonols, was considered to figure out the preliminary mechanism because of its ability to attract metals due to the presence of phenolic groups [34].

**Scheme 1:**
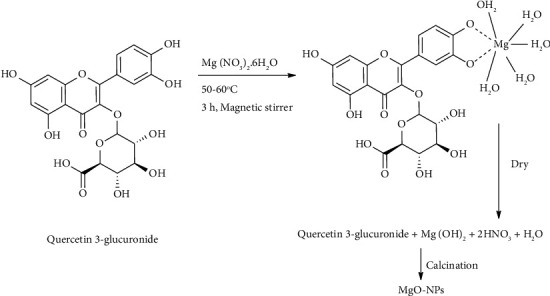
Tentative mechanism for production of nanoparticles using extracts.

The biosynthesis of MgO-NPs from the strawberry seed extract is depicted in Scheme 1 Flavonoids engage metal nitrate through weak bonding to form a complex, such as the metal-flavonoid complex shown in Scheme 1. After 8 hours in a hot air oven, the complex solution is transformed into hydroxide forms. Calcination is employed in the final step to produce metal oxide nanoparticles from biosynthesized hydroxide complexes [29]. Thus, in this mechanism, the bioactive molecules of flavonoids play an important role in the hydrolysis process and aid in the formation of NPs by acting as caps.

### 3.6. Dynamic Light Scattering (DLS)

**Figure 9:**
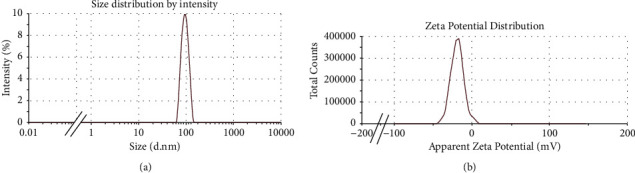
(a) Size distribution by the intensity graph of MgO-NPs and (b) zeta potential for MgO-NPs.

To study the particle size distribution and zeta potential (*ζ*) of nanomaterials dispersed in solution or colloidal suspensions, dynamic light scattering (DLS) is considered to be an efficient and statistically reliable method. Biosynthesized MgO-NPs dispersed in water were analyzed for particle size using the dynamic light scattering principle with Zetasizer Nano ZS, Malvern Instruments. The DLS result from Figure 9(a) showed that the hydrodynamic diameter of the particle size distribution of MgO-NPs was greater than 119 nm with a polydispersity index (PDI) of 0.47. The size of the particles is usually larger than that which is measured with other macroscopic techniques, such as TEM (average diameter of 100 nm for MgO-NPs in the present study), due to the influence of Brownian motion, in addition to including the hydrodynamic diameter in the calculation of particle size [35]. The obtained single peak indicated that the quality of the biosynthesized NPs was satisfactory. A common method for determining the stability of a colloidal system is to use zeta potential (*ζ*) to determine the surface charge of particles. MgO-NPs in distilled water had a zeta potential of -34.5 mV, specifying the stability of the colloidal solution (Figure 9(b)). It has been determined that suspensions with a voltage of 15 mV are able to support stable colloids [36].

### 3.7. Antibacterial Activity

**Figure 10:**
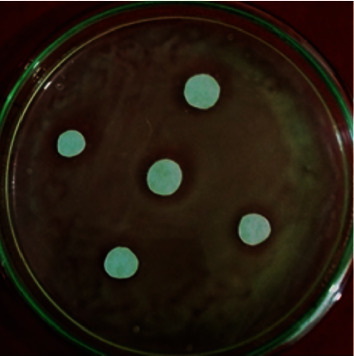
In vitro inhibition zone around the paper disc treated with different concentrations of MgO-NPs.

**Figure 11:**
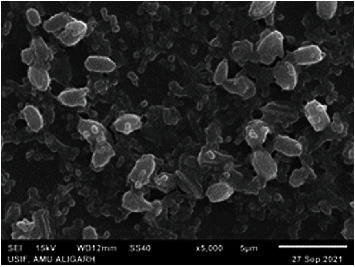
SEM micrograph of bacteria treated with MgO-NPs.

The values of the zone of inhibition for the MgO-NPs obtained in *in vitro* evaluation against *R. solanacearum* (Table 1 and Figures 10 and 11) are given in millimeters (mm). The antibacterial activities of MgO-NPs were also compared to controls lacking any type of MgO. The inhibition zone was absent in the control. Using a broth dilution procedure, the MIC was also calculated as the lowest concentration. The MIC was determined to be 10 *μ*g/mL, which was the lowest concentration with a clear inhibitory zone. SEM analysis showed the disturbing bacterial cells after being treated with MgO-NPs (Figure 11). MgO-NPs can be used to manage plant diseases caused by pathogens [37]. Magnesium oxide nanoparticles (MgO-NPs) show antimicrobial activity. The antibacterial and antifungal properties of MgO NPs have been previously cited in various kinds of the literature. Nanosized MgO surprisingly caused more inhibition of conidial germination in *R. stolonifer*, *M. plumbeus*, *A. alternata*, *and F. oxysporum* as compared to nanoscaled zinc oxide [38].

### 3.8. MgO-NPs' Effect on Egg Hatching and Mortality under *In Vitro*

**Figure 12:**
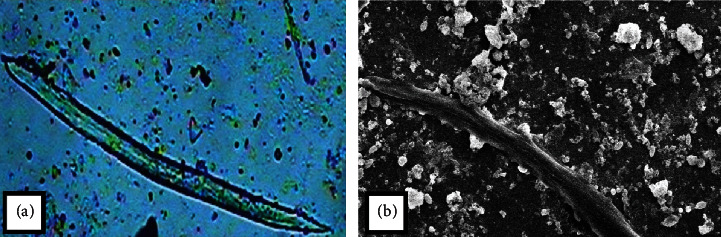
(a) Microscopic images of the treated nematode and (b) SEM images of the treated nematode.

Direct exposure of MgO-NPs to *M. incognita* in water showed a toxic effect on J2 of *M. incognita* and causes mortality (Table 2). On increasing the exposure time and concentration, the effect became more prominent. Egg hatching is also inhibited by MgO-NPs. The maximum hatching occurred at 24 and 48 hours in double distilled water (DDW). The least number of dead nematodes was observed in DDW after 48 hours. Based on MgO-NPs' applications, the egg hatching was found to be decreasing with increasing time. Figures 12(a) and 12(b) show microscopic and scanning electron microscopy images of the nematode treated with MgO nanoparticles, demonstrating morphological changes in the nematode.

### 3.9. Antioxidant Capacity Analysis

The ability of nanoparticles to scavenge free radicals at varying concentrations was assessed using DPPH assays. The free radical DPPH is neutralized by absorbing hydrogen from a hydrogen donor molecule or electron transfer nanoparticles. The violet color of DPPH fades when it is reduced, suggesting the presence of free radical scavenging nanoparticles in the reaction mixture.

The MgO-NPs synthesized from the strawberry seed extract are possible free radical scavengers with an effective dose-dependent inhibition activity. Different concentrations of MgONP 75, 150, 300, and 500 *μ*g/ml scavenged DPPH by 35.77, 45.56, 50.23, and 55.20%, respectively. However, these capacities are inferior to those of ascorbic acid, the reference standard used (Table 3). As per Table 3, MgO-NPs have a lower DPPH scavenging capacity with an IC_50_ of 278.9 *μ*g/mL than ascorbic acid (IC_50_ of 35.3 *μ*g/mL)

## 4. Discussion

This study demonstrates the antinematode behavior of MgO-NPs under *in vitro* conditions. This finding reaffirms the earlier reports, which documented the bactericidal effect of MgO-NPs against some Gram-negative and Gram-positive bacteria [39]. Xin et al. [4] found that MgO-NPs showed bactericidal activity against *E. coli* and *S. aureus*. However, the exact process behind the bactericidal action of MgO-NPs is not known, and Leung et al. [40] suggested that attachment of NPs combined with the change in pH and release of Mg^2+^ ions might result in membrane damage. Root-knot nematodes (RKNs) are the most destructive PNs worldwide. The second-stage RKN juvenile (J2) is infective, which causes perforation and initiation of giant cell formation in roots. The stylet of PNs helps penetrate the root cell wall, and earlier reports hint at the existence of virulence effector proteins, cell wall degrading, and cell wall modifying enzymes, namely, pectate lyases, *β*-1,4-endoglucanases, expansins, and polygalacturonases, and transcription factors inside the stylet secretome [21, 41]. MgO, being a solid-base catalyst, can trigger deprotonation (acceptor of hydrogen atom) [42]. Hence, ROS generation in MgO-NP-treated brinjal roots might have occurred due to deprotonation of phenolic hydroxyls resulting in phenoxyl radicals. Generally, a prominent increase in ROS is a resistance reaction to pathogen attacks in plants. A rapid ROS generation in presence of *R. solanacearum* was noticed in the resistant cultivar of tomato, i. e., BT-10 [43]. In conclusion, the results of this study have shown that MgO-NPs might suppress root-knot and R. solanacearum in plants. As can be seen from Table 3, as the concentration of nanoparticles (75, 150, 300, and 500 *μ*g/ml) increases, so does the percentage of the inhibitor capacity (35.77, 45.56, 50.23, and 55.20%), demonstrating that MgoNPs are associated with DPPH in the reaction. However, the activity of nanoparticles was inferior to that of the standard compound ascorbic acid (Table 3).

## 5. Conclusions

Our findings give a holistic and alternative approach to the synthesis of MgO-nanoparticles using the plant material that is efficient and eco-friendly. The biosynthesized nanoparticles of the present paper can inhibit bacterial growth and have also demonstrated nematicidal activity as well as antioxidant action against free radicals in a concentration-dependent way. With these findings, we could conclude that MgO-NPs could be used to manage plant pathogenic bacteria *R. solanacearum* and root-knot nematode *M. incognita*.

## Figures and Tables

**Table 1:** Antibacterial activity of MgO-NPs against the pathogenic strain of *R. solanacearum* expressed as an inhibition diameter zone in millimeters (mm).

MgO-NP concentration	Test organism	Zone of inhibition in the presence of MgO-NPs (mm)	Zone of inhibition in the absence of MgO-NPs (mm)
10 *μ*g/ml	*R. solanacearum*	4 ± 0.2	0
20 *μ*g/ml	*R. solanacearum*	6 ± 0.2	0
30 *μ*g/ml	*R. solanacearum*	9 ± 0.4	0
40 *μ*g/ml	*R. solanacearum*	12 ± 0.4	0
50 *μ*g/ml	*R. solanacearum*	15 ± 0.5	0

**Table 2:** Effect of NPs on *M. incognita* hatching and mortality.

Treatment	Hatching of J2of *M. incognita*	Mortality of J2		
	24 hours	48hours	24hours	48hours
Distilled water	94 ± 3	217 ± 3	0	3
50 *μ*g/ml MgO-NPs	85 ± 2	194 ± 2	1	5
100 *μ*g/ml MgO-NPs	71 ± 2	106 ± 2	04	14

**Table 3:** DPPH assay to measure the antioxidant activity of NPs at different concentrations.

NPs and reference	Concentration (*μ*g/mL)				IC50 (*μ*g/mL)
	75	150	300	500	
	% Inhibition				
MgO-NPs	35.77	45.56	50.23	55.20	278.9
Ascorbic acid	40.59	52.57	62.36	75.80	35.3

## Data Availability

The data used to support the findings of this study are included within the article.
